# Amblyopia Produced by Tobacco Smoking

**Published:** 1867-09

**Authors:** 


AMBLYOPIA PRODUCED BY TOBACCO SMOKING.—M. Viardin has reported three cases of amblyopia caused by smoking. In the treatment of these cases the quantity of tobacco smoked was reduced under the direction of M. Viardin, and the sight was restored in the course of a few weeks.

